# Cognitive flexibility moderates the relationship between exposure to COVID stressors and obsessive-compulsive (OCS) symptoms

**DOI:** 10.1192/j.eurpsy.2023.531

**Published:** 2023-07-19

**Authors:** L. Albertella, C. Liu, R. S. C. Lee, L. Fontenelle, S. R. Chamberlain, M. Yücel, K. Rotaru

**Affiliations:** ^1^Turner Institute for Brain and Mental Health, Monash University, Melbourne, Australia; ^2^Obsessive, Compulsive, and Anxiety Spectrum Research Program, Institute of Psychiatry, Federal University of Rio de Janeiro, Rio de Janeiro, Brazil; ^3^Department of Psychiatry, University of Southampton, Southampton, United Kingdom; ^4^Monash Business School, Monash University, Melbourne, Australia

## Abstract

**Introduction:**

Research suggests that the COVID-19 pandemic and related stressors have triggered OCS for many individuals. However, the extent to which the pandemic and related stressors have influenced OCS seems to vary by individual factors, with some individuals being at greater risk than others. Despite the well-known role of cognitive inflexibility as a marker of risk for OCS, no study to date has examined the extent to which it influences individual susceptibility to developing OCS during the current pandemic. Toward this aim, the current study examined whether cognitive flexibility moderates whether exposure to COVID-related stressors is associated with OCS. Research suggests that the COVID-19 pandemic and related stressors have triggered OCS for many individuals. However, the extent to which the pandemic and related stressors have influenced OCS seems to vary by individual factors, with some individuals being at greater risk than others. Despite the well-known role of cognitive inflexibility as a marker of risk for OCS, no study to date has examined the extent to which it influences individual susceptibility to developing OCS during the current pandemic.

**Objectives:**

Toward this aim, the current study examined whether cognitive flexibility moderates whether exposure to COVID-related stressors is associated with OCS.

**Methods:**

Participants were 169 students (age = 22 years, 62% female) from two student cohorts at Monash Business School who reported experiencing current OCS symptoms. All cohorts completed an online visual search task to measure flexibility of reward-related attentional capture (as an index of cognitive flexibility; measured using the VMAC-R task) and questionnaires gauging exposure to COVID-related stressors, pre-pandemic OCS, and current/lockdown OCS. A negative binomial regression examined the extent to which a) number of COVID-related stressors, b) cognitive flexibility, and c) their interaction was associated with lockdown OCS, adjusting for pre-COVID OCS.

**Results:**

The interaction between COVID-related stressors and cognitive flexibility was significantly associated with OCS (*p* = 0.048). Follow-up analyses showed that this interaction was driven by exposure to COVID-related stressors being associated with greater OCS among individuals with high cognitive inflexibility scores only (*p* = .029). Among cognitively flexible individuals, we did not find a relationship between COVID-related stressors and OCS (*p* = .470).

**Conclusions:**

The result of this study highlight the role of cognitive flexibility as a potential moderator between COVID events and OCS. Critically, these findings have implications for detecting who is at risk of developing OCS following exposure to COVID-related stressors, and suggest that future interventions aimed at modifying cognitive flexibility may hold promise for boosting resilience against the effects of COVID-related stressors on OCS.

**Disclosure of Interest:**

None Declared

